# Intracellular Accumulation of Methylglyoxal by Glyoxalase 1 Knock Down Alters Collagen Homoeostasis in L6 Myoblasts

**DOI:** 10.3390/ijms18030480

**Published:** 2017-02-23

**Authors:** Bernd Stratmann, Bernhard Goldstein, Paul J. Thornalley, Naila Rabbani, Diethelm Tschoepe

**Affiliations:** 1 Herz- and Diabeteszentrum NRW, Diabeteszentrum, Ruhr Universität Bochum, 32545 Bad Oeynhausen, Germany; bgoldstein@hdz-nrw.de (B.G.); diethelm.tschoepe@rub.de (D.T.); 2 Warwick Medical School, Clinical Sciences Research Laboratories, University of Warwick, University Hospital, Coventry CV2 2DX, UK; P.J.Thornalley@warwick.ac.uk (P.J.T.); N.Rabbani@warwick.ac.uk (N.R.)

**Keywords:** hyperglycemia, dicarbonyl proteome, methylglyoxal, collagen, glyoxalase 1

## Abstract

Hyperglycemia results in accumulation of the reactive dicarbonyl methylglyoxal (MG). Methylglyoxal is detoxified by the glyoxalase system (glyoxalase 1 and 2). The influence of glyoxalase 1 knockdown on expression of collagens 1, 3, 4, and 5 in L6 myoblasts under hyperglycemic conditions was investigated. Increased biosynthesis of collagens 1, 3, 4, and 5 was detected at mRNA-level following knockdown of glyoxalase 1 (GLO1). At the protein level a significant elevation of the concentration of collagen 1 and 4 was shown, whereas no increase of collagen 5 and a non-significant increase in collagen 3 were detectable. These results could partially explain MG-induced changes in the extracellular matrix (ECM) which account for increased fibrosis and impaired function in myocytes. The mechanisms by which reactive glucose metabolites influence ECM composition deserve further investigation.

## 1. Introduction

Diabetes mellitus is a metabolic disorder characterized by chronic hyperglycemia. The increased plasma glucose arising from increased insulin resistance or insulin deficiency and metabolic impairment leads to increased formation and accumulation of the highly reactive dicarbonyl methylglyoxal (MG) resulting in dicarbonyl stress [1,2,3]. The reaction of MG with macromolecules, such as proteins, DNA, and basic phospholipids leads to the formation of advanced glycation end products (AGEs), which are likely involved in the pathogenesis of vascular complications, nephropathy, retinopathy, neuropathy, and increased risk of cardiovascular and cerebrovascular disease [4,5,6,7]. MG is formed mainly by the trace degradation of triosephosphates, glyceraldehyde-3-phosphate (GA3P), and dihydroxyacetone phosphate (DHAP) with normally minor contributions from metabolism of ketone bodies, threonine catabolism, and degradation of glycated proteins [8]. Detoxification of MG is mediated by the glutathione-dependent glyoxalase system consisting of the enzymes glyoxalase 1 and glyoxalase 2, and a catalytic amount of reduced glutathione (GSH) [9]. Glycation by MG imposes structural and potentially functional modifications. Formation of protein-derived AGEs causes structural distortion, loss of side chain charge of arginine residues, enzyme inactivation, and other protein dysfunction [10,11]. Examples of proteins susceptible to MG modification and functional impairment, called the “dicarbonyl proteome” (DCP), are proteins of the extracellular matrix (ECM)—collagen, fibronectin, and laminin [12,13]—and mitochondrial proteins [14]. ECM proteins often present a higher content of AGE residues than other proteins modified due to their long half-life. AGEs may influence several signaling pathways, including inflammation through the effect on the receptor for AGEs (RAGE) and its ligands, hypoxia-related responses through modification of p300, and others [15,16]. Dicarbonyl stress may be both the cause and consequence of oxidative stress as MG modification of mitochondrial proteins stimulates increased reactive oxygen species (ROS) formation. Depletion of Glutathion (GSH) and Nicotinamide Adenine Dinucleotide Phosphate Hydrogen (NADPH) decreases the in situ activity of glyoxalase 1 (GLO1) and aldehyde dehydrogenases leading to MG and the accumulation of other dicarbonyl metabolites [17]. Oxidative stress increases glucose uptake and stimulates glucose transporter 4 (GLUT4)-translocation in adipocytes and muscle cells [18,19]. Insulin-dependent GLUT4 is responsible for insulin-regulated glucose uptake into fat and muscle cells to prevent hyperglycemia. Under steady state conditions GLUT4 is continuously relocating between intracellular compartments and the plasma membrane; 2%–5% are located on the cell surface and the main part of GLUT4 resides in intracellular GLUT4 storage vesicles (GSVs). After insulin-induction, plasma membrane levels of GLUT4 raise 5- to 30-fold [20]. Our previous study revealed an increased internalization of glucose depending on prolonged GLUT4 translocation on the plasma membrane, which is not related to increased oxidative stress or insulin action [21].

In this study, we further investigated the influence of dicarbonyl stress on the composition and regulation of the ECM in a study of rat L6 myoblasts by employing a GLO1-specific knockdown under conditions of high glucose concentration to simulate hyperglycemia. We have chosen this model because the MG induced pronounced glucose influx has been studied and proven in these cells before as being independent from GLUT4 expression level and oxidative stress, suggesting a new MG dependent mechanism of exaggerated glucose uptake [21,22]. As the GLUT4 translocation and the increased glucose influx can easily be determined in these cells, they represent an adequate model for further studies on the consequences of glucose and MG overflow. Having the glucose intoxication hypothesis in mind, we wanted to determine the direct consequences on the expression profile on collagens 1, 3, 4, and 5 to evaluate the contribution of MG and the associated pronounced glucose influx on collagen expression as markers of fibrotic remodeling processes. Our results revealed alterations in the biosynthesis of fibrillary and fibril-associated collagens leading to possible remodeling of the ECM, thereby indicating induction of fibrotic processes.

## 2. Results

### 2.1. Knockdown of GLO1

**Figure 1:**
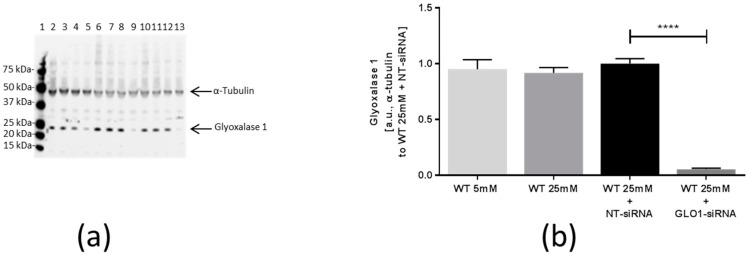
Analysis of glyoxalase 1 (GLO1) expression. (**a**) Western Blot analysis for GLO1 protein of L6 myoblasts transfected with non-target siRNA and GLO1 specific siRNA following 48 h siRNA transfection. Lane 1: Precision Plus Protein Standard; lane 2, 6, 10: WT 5 mM; lane 3, 7, 11: WT 25 mM, lane 4, 8, 12: WT 25 mM + non-target siRNA (NT-siRNA); lane 5, 9, 13: WT 25 mM + GLO1-siRNA. (**b**) Graphical analysis of Western Blot. Data are mean ± SEM (*N* = 3, *n* = 3). Significance: **** *p* < 0.0001, WT 25 mM + GLO-siRNA vs. WT 25 mM + NT-siRNA.

After knockdown of GLO1, the impact of intracellular accumulation of MG in L6 myoblasts on the expression of collagens and collagen-modifying enzymes was analyzed. Therefore, a knockdown of GLO1 was conducted by transfection of the wildtype (WT) cells with specific GLO1 directed siRNA (WT 25 mM + GLO1-siRNA) and non-target siRNA (WT 25 mM + non-target siRNA (NT-siRNA)) as control. Transfected and non-transfected cells (WT 25 mM) were incubated under hyperglycemic conditions (25 mM glucose in culture-medium) for 48 h. Additionally, non-transfected cells (WT 5 mM) were incubated under normoglycemic conditions (5 mM glucose), to reveal possible expressional changes induced by hyperglycemic conditions. The GLO1 specific knockdown resulted in 90% down regulation of glyoxalase 1 on protein level (0.06 ± 0.02 vs. 1.00 ± 0.12 arbitrary units (a.u.), *p* < 0.0001, WT 25 mM + GLO1-siRNA vs. WT 25 mM + NT-siRNA) (Figure 1). As already detected before [21], GLO1 knockdown resulted in a prolonged translocation of GLUT4 to the cell surface, an increased glucose influx, and pronounced apoptosis.

### 2.2. Accumulation of Methylglyoxal, Glyoxal, and Related Advanced Glycation Endproducts

**Figure 2:**
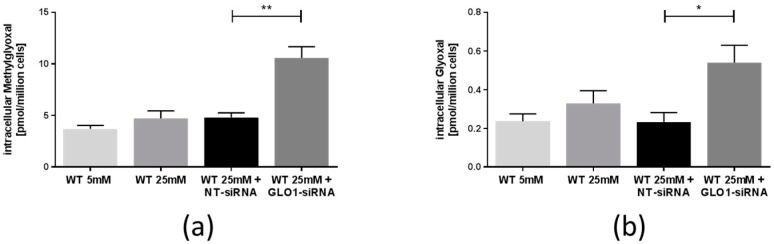

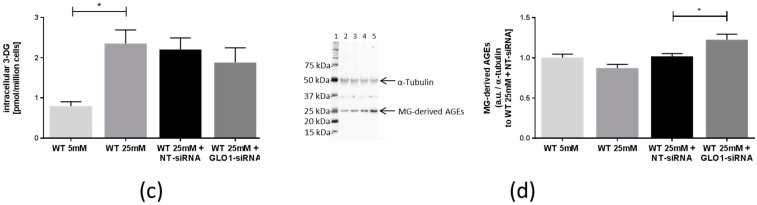
Analysis of formation of methylglyoxal (MG), glyoxal, 3-deoxyglucosone (3-DG), and methylglyoxal-derived advanced glycation end products (AGEs). (**a**) MG content of L6 myoblasts transfected with non-target siRNA and GLO1 specific siRNA following 48 h siRNA transfection. Data are mean ± SEM (*N* = 3, *n* = 3). Significance: ** *p* < 0.01, WT 25 mM + GLO-siRNA vs. WT 25 mM + NT-siRNA; (**b**) glyoxal content of L6 myoblasts transfected with non-target siRNA and GLO1 specific siRNA following 48 h siRNA transfection. Data are mean ± SEM (*N* = 3, *n* = 3). Significance: * *p* < 0.05, WT 25 mM + GLO-siRNA vs. WT 25 mM + NT-siRNA; (**c**) 3-DG content of L6 myoblasts transfected with non-target siRNA and GLO1 specific siRNA following 48 h siRNA transfection. Data are mean ± SEM (*N* = 3, *n* = 3). Significance: * *p* < 0.05, WT 25 mM vs. WT 5 mM; (**d**) Western Blot analysis for MG-derived AGE content of L6 Myoblasts transfected with non-target siRNA and GLO1 specific siRNA following 48 h siRNA transfection. Lane 1: Precision Plus Protein Standard; lane 2: WT 5 mM; lane 3: WT 25 mM, lane 4: WT 25 mM + NT-siRNA; lane 5: WT 25 mM + GLO1-siRNA; Data are mean ± SEM (*N* = 3, *n* = 3). Significance: * *p* < 0.05, WT 25 mM + GLO-siRNA vs. WT 25 mM + NT-siRNA.

The cellular concentrations of MG, glyoxal, and 3-deoxyglucosone (3-DG) were determined by LC-MS/MS [23]. The results showed a 2.2-fold increase in the cellular concentration of MG with GLO1 knockdown, compared to non-target siRNA transfected control (10.57 ± 1.10 vs. 4.81 ± 0.45 pmol/106 cells, equivalent to a cellular concentration increase of MG from 2.9 to 6.4 µM in L6 cells with GLO1 knockdown (assuming a L6 cell volume of 1.67 µL per 106 cells)) (Figure 2a). Concomitantly, after GLO1 knockdown, glyoxal concentration increased 2.3-fold (0.54 ± 0.09 vs. 0.23 ± 0.05 pmol/106 cells (Figure 2b). 3-DG concentrations did not vary upon GLO1 knockdown, but increased significantly with hyperglycemia (Figure 2c). Accumulation of MG-derived AGEs was measured by Western blot analysis. MG-derived AGEs were significantly enhanced by 20% in cells with GLO1 knockdown compared to the NT-siRNA (1.23 ± 0.07 vs. 1.00 ± 0.06 a.u., *p* < 0.05, WT 25 mM + GLO1-siRNA vs. WT 25 mM + NT-siRNA) (Figure 2d).

### 2.3. Biosynthesis of Collagens

**Figure 3:**
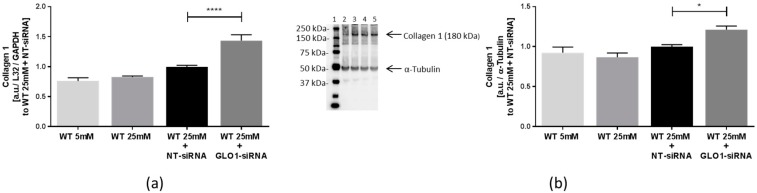
Collagen 1 expression in L6 myoblasts transfected with non-target siRNA and GLO1 specific siRNA. (**a**) Collagen 1 mRNA; (**b**) collagen 1 protein. L6 myoblasts were transfected with non-target siRNA and GLO1 specific siRNA following 48 h siRNA transfection. Lane 1: Precision Plus Protein Standard; lane 2: WT 5 mM; lane 3: WT 25 mM, lane 4: WT 25 mM + NT-siRNA; lane 5: WT 25 mM + GLO1-siRNA. Data are mean ± SEM (*N* = 3, *n* = 3). Significance: * *p* < 0.05 and **** *p* < 0.0001, WT 25 mM + GLO-siRNA vs. WT 25 mM + NT-siRNA.

To examine the influence of GLO1 knockdown on the composition of the ECM, we analyzed the biosynthesis of dominant expressed collagen 1 in muscle tissues, as well as collagen 1-associated collagens by real-time PCR and Western blot. Our results showed an increased collagen-biosynthesis in cells with GLO1 knockdown. As a part of striated fibrils, collagen 1 is highly expressed in muscle tissues. After GLO1 knockdown, collagen 1 mRNA was increased (1.43 ± 0.10 vs. 1.00 ± 0.02 a.u., *p* < 0.0001, WT 25 mM + GLO1-siRNA vs. WT 25 mM + NT-siRNA) and collagen 1 protein was also increased (1.21 ± 0.11 vs. 1.00 ± 0.07 a.u., *p* < 0.05, WT 25 mM + GLO1-siRNA vs. WT 25 mM + NT-siRNA) (Figure 3a,b).

**Figure 4:**
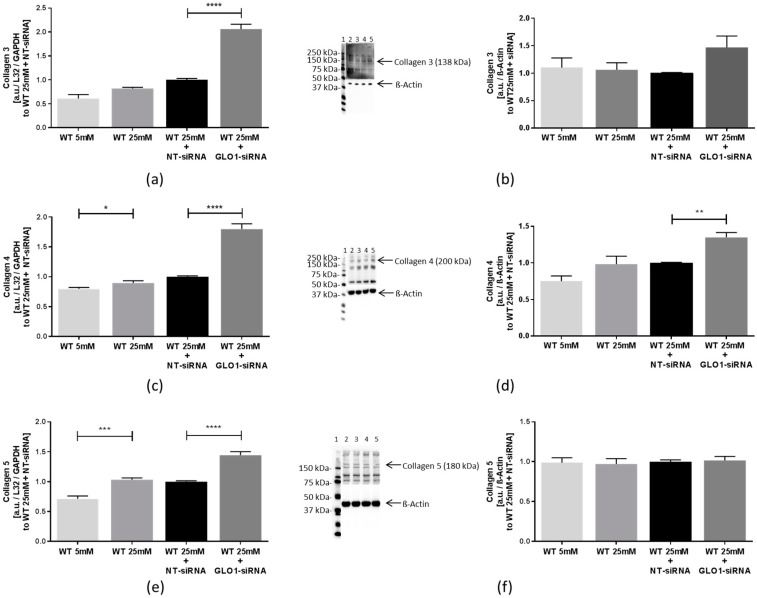
Expression of collagen 3, 4, and 5 in L6 myoblasts transfected with non-target siRNA and GLO1 specific siRNA. L6 Myoblasts were transfected with non-target siRNA and GLO1 specific siRNA following 48 h siRNA transfection. (**a**) Collagen 3 mRNA; (**b**) collagen 3 protein. Lane 1: Precision Plus Protein Standard; lane 2: WT 5 mM; lane 3: WT 25 mM, lane 4: WT 25 mM + NT-siRNA; lane 5: WT 25 mM + GLO1-siRNA; (**c**) collagen 4 mRNA; (**d**) collagen 4 protein. lane 1: Precision Plus Protein Standard; lane 2: WT 5 mM; lane 3: WT 25 mM, lane 4: WT 25 mM + NT-siRNA; lane 5: WT 25 mM + GLO1-siRNA; (**e**) collagen 5 mRNA, and (**f**) collagen 5 protein. lane 1: Precision Plus Protein Standard; lane 2: WT 5 mM; lane 3: WT 25 mM, lane 4: WT 25 mM + NT-siRNA; lane 5: WT 25 mM + GLO1-siRNA. Data are mean ± SEM (*N* = 3, *n* = 3). Significance: * *p* < 0.05, ** *p* < 0.01, *** *p* < 0.001, and **** *p* < 0.0001, WT 25 mM + GLO-siRNA vs. WT 25 mM + NT-siRNA, or WT 25 mM vs. WT 5 mM, respectively.

Collagen 1-associated collagen 3 mRNA was also increased approximately 2-fold in cells with GLO1 knockdown, compared to the NT-siRNA treated cells (2.06 ± 0.10 vs. 1.00 ± 0.03 a.u., *p* < 0.0001, WT 25 mM + GLO1-siRNA vs. WT 25 mM + NT-siRNA) and collagen 3 protein was also, but non-significantly, increased (1.47 ± 0.21 vs. 1.00 ± 0.01 a.u., *p* > 0.05, WT 25 mM + GLO1-siRNA vs. WT 25 mM + NT-siRNA) (Figure 4a,b). High glucose concentration produced modest increases of collagen 4 mRNA (1.03 ± 0.03 vs. 0.71 ± 0.05 a.u., *p* < 0.001, WT 25 mM vs. WT 5 mM); knockdown of GLO1 further increased the levels of collagen 4 mRNA. Collagen 4 mRNA was increased 1.8-fold in cells with GLO1 knockdown, compared to the NT-siRNA treated cells (1.80 ± 0.09 vs. 1.00 ± 0.01 a.u., *p* < 0.0001, WT 25 mM + GLO1-siRNA vs. WT 25 mM + NT-siRNA) (Figure 4c) and collagen 4 protein was also increased (1.35 ± 0.07 vs. 1.00 ± 0.01 a.u., *p* < 0.01, WT 25 mM + GLO1-siRNA vs. WT 25 mM + NT-siRNA) (Figure 4d). Fibrillary collagen 5 mRNA was increased by hyperglycemia (1.03 ± 0.03 vs. 0.71 ± 0.05 a.u., *p* < 0.001, WT 25 mM vs. WT 5 mM) and 1.4-fold elevated by GLO1 knockdown (1.44 ± 0.06 a.u. vs. 1.00 ± 0.01 a.u., *p* < 0.0001, WT 25 mM + GLO1-siRNA vs. WT 25 mM + NT-siRNA), whereas collagen 5 protein was unchanged (Figure 4e,f).

## 3. Discussion

Our previous study revealed an intracellular accumulation of MG as well as enhanced glucose internalization after knockdown of GLO1 in cells under hyperglycemic conditions [21]. These findings may be in relation to the fibrotic processes associated with diabetes in myoctes, as exaggerated intracellular concentrations of glucose have been shown to enhance ECM accumulation and MG has been proven to modify existing proteins in terms of irreversible crosslinking [24]. The aim of this study was to analyze the consequences of a MG driven enhanced glucose uptake on the expression of several structurally related collagens as main components of the extracellular matrix.

Under low glucose conditions, the insulin-dependent glucose transporter 4 (GLUT4) continuously relocates between cell-surface and intracellular storage-vesicles [20]. An insulin-mediated increase of GLUT4 concentration on the cell-surface enhances glucose-uptake to prevent an extracellular hyperglycemic milieu and to deliver glucose for metabolic purposes. In our cell culture model, we revealed an impaired GLUT4 internalization leading to the prolonged presentation of GLUT4 on the cell-surface, resulting in increased glucose-uptake after knockdown of GLO1 [21]. Increased GLUT4-level on the cell-surface after GLO1 knockdown or extracellular MG stimulation in L6 cells appeared unrelated to MG modification or expression of GLUT4 and oxidative stress [21,22]. These results suggest that MG may influence mechanisms involved in GLUT4-trafficking and subsequent glucose uptake. The aim of this study was to further investigate the influence of GLO1 knockdown and MG accumulation on the regulation and composition of the ECM in this well-defined cell culture model.

After silencing of GLO1, there was an approximately 2.2-fold increase in MG and a 2.3-fold increase in glyoxal concentration and increased MG-derived AGEs; 3-DG concentration did not vary upon knockdown of GLO1. This suggests that GLO1 silencing is effective and imposes dicarbonyl stress in L6 cells. Glycation caused by MG may impair cell homeostasis by altering functions of crucial enzymes of metabolic pathways. With regard to the ECM, we could detect an increased biosynthesis of fibrillary and fibril-associated collagens (collagens 1–5) after GLO1 knockdown, suggesting a remodeling of the ECM, which in turn, displays the induction of fibrotic processes. Furthermore, increased biosynthesis of collagen 1 was identified as an indicator for the onset of fibrotic processes [25]. The observed effects are attributable to the accumulation of MG during hyperglycemic culture conditions and the consequences arising from increased MG concentrations, as—in the control experiments—hyperglycemia alone was analyzed in comparison to normoglycemia, and GLO1 knockdown experiments were performed in addition to hyperglycemia. Hyperglycemia alone was not able to induce the effects seen with GLO1 knockdown.

Accumulation of MG in cells results in increased production of ROS and oxidative stress [8,26,27], which in turn may induce further glucose uptake and GLUT4-translocation [18,19,28,29]. In our model, increased glucose uptake is not dependent on oxidative stress, but MG dependent [22]. Similarly, increased biosynthesis of collagens induced by GLO1 knockdown may not be regulated by oxidative stress, particularly as protein modification by MG is non-oxidative. By coincubation with the antioxidant tiron we were able to show that the collagen production is not related to the increase in oxidative stress. Increased collagen synthesis is characteristic of L6 cells as they approach confluence and it decreases thereafter [30]. Knockdown of GLO1 may increase MG modification of collagens and alter functional contact with cells, as shown for cardiac fibroblasts cultured on MG-modified collagen matrices and for MG-modifications of collagen 1 and collagen 4 [12,31,32]. Consequences of the observed alterations in collagen concentration as being responsible for disturbed cell-matrix contact contributing to apoptotic processes in our model is speculative at this time point and deserves further analysis.

## 4. Materials and Methods

### 4.1. Cell Culture

L6-GLUT4-myc-tagged rat myoblasts were used as a cell culture model [33]. L6-GLUT4-myc cells were maintained in α-minimal essential medium (α-MEM, Invitrogen, Carlsbad, CA, USA) containing 5 mM glucose, supplemented with 10% (*v*/*v*) fetal bovine serum (FBS, PAA Laboratories, Coelbe, Germany) and antimycotic/antibiotic solution (100 units/mL penicillin, 100 µg/mL streptomycin, 250 ng/mL amphotericin B; Invitrogen, Carlsbad, CA, USA) at 37 °C in a humidified atmosphere of 5% CO_2_. Cells were grown to 70%–80% confluence.

### 4.2. Gene Specific Knockdown Using siRNA

For transient transfection L6-GLUT4myc cells were grown in six-well plates at a density of 1 × 10^5^ cells per well. Cells were transfected with 75 nM siRNA (final concentration) directed against GLO1 (Sequence: sense: 5′-CCA AGG AUU UUC UAC UGC Utt-3′, antisense: 5′-UGC AGU AGA AAA UCC UGG Gtg-3′, Ambion, Austin, TX, USA) or with a non-target siRNA as control, using Lipofectamine^TM^ 2000 transfection reagent (Invitrogen, Carlsbad, CA, USA) according to the manufacturer’s instructions. Next, 3 µL of siRNA (50 µM) and 3 µL Lipofectamine^TM^ 2000 were diluted with 247 µL Opti-MEM, and incubated for 20 min at room temperature, and then added to the cells. Subsequently, 1.5 mL transfection-medium (α-MEM with 10% FBS but without antibiotic) were added to each culture well. Twenty-four hours after transfection, the medium was replaced by fresh complete medium with 25 mM glucose or 5 mM glucose, respectively. As experimental controls, cells were incubated under unchanged normoglycemic (5 mM glucose in culture medium) and hyperglycemic (25 mM glucose in culture medium) conditions. For siRNA-control, cells were transfected with non-target siRNA (NT-siRNA) and incubated under hyperglycemic conditions. Assays were done 48 h post-transfection.

### 4.3. Immunoblotting Analysis

Cells were washed twice with ice-cold PBS and lysed using lysis buffer containing sodium chloride (150 nM), Tris-HCL (50 nM), NaF (1 mM), EDTA (1 mM), Na_3_VO_4_ (1 mM), Nonidet P40 (1%), sodium deoxycholate (0.25%), and proteinase inhibitor cocktail (Sigma-Aldrich, Saint Louis, MO, USA); the cells were repeatedly frozen, and were finally lysed by sonication. The total protein concentrations were determined by bicichoninic acid assay (Sigma-Aldrich, Saint Louis, MO, USA) using bovine serum albumin (BSA) for a standard curve. Proteins were separated by NuPAGE^®^ 4%–12% Bis-Tris gel (Invitrogen, Carlsbad, CA, USA) electrophoresis and electrotransferred (Trans-blot SD, Biorad, Hercules, CA, USA) to nitrocellulose membranes (Biorad, Hercules, CA, USA). Precision Plus Protein Standard (BioRad, Cat. No. 161-0374) was applied to serve as a molecular weight marker. Membranes were incubated for 1 h in blocking-solution containing 5% dry milk in Tris-buffered saline. Membranes were washed in Tris-buffered saline and incubated overnight at 4 °C with the following antibodies: monoclonal anti-GLO1 (1:1000; Abcam, Cambridge, UK), monoclonal anti-MG-derived AGE (1:1000; NoF Corporation, Tokyo, Japan), monoclonal anti-collagen 1 (1:1000; LifeSpan BioSciences, Seattle, WA, USA), polyclonal anti-collagen 4 (1:1000; Abcam, Cambridge, UK), and monoclonal anti-collagen 5 (1:1000; Abnova, Taipei, Taiwan). Collagen 3 was measured in cell culture supernatant using polyclonal anti-collagen 3 (1:1000; Abcam, Cambridge, UK). For detection of reference proteins monoclonal anti-β-actin (1:1000; Cell Signaling Technology, Danvers, MA, USA) and monoclonal anti-α-tubulin (1:1000; Cell Signaling Technology, Danvers, MA, USA) were used. After washing, the membranes were incubated for 1 h with horseradish Peroxidase (HRP)-conjugated secondary antibodies. As a HRP substrate Chemiglow (Alpha Innotech Corporation, San Leandro, CA, USA) was used and recorded by a Charge Coupled Device (CCD)-camera system (Flour Chem FC3, CellBiosciences, Santa Clara, CA, USA).

### 4.4. Quantitative Real Time-PCR Analysis (qRT-PCR)

Cells were washed twice with ice-cold PBS and detached using RLT buffer containing β-mercaptoethanol (10 µL per mL) (Qiagen, Hilden, Germany). After cell lysis using QIAshredder columns (Qiagen, Hilden, Germany), RNA was purificated with RNeasy Mini Kit (Qiagen, Hilden, Germany). RNA concentrations were determined by NanoDrop 2000 (PeqLab Biotechnologie GmbH, Erlangen, Germany). After reverse transcription, purified cDNA was analyzed via quantitative real time-PCR using MasterCycler^®^ ep realplex (Eppendorf, Hamburg, Germany) using Platinum^®^ SYBR^®^ Green qPCR SuperMix-UDG (Invitrogen, Carlsbad, CA, USA). Oligonucleotide sequences are available upon request.

### 4.5. Chromatographic and Mass Spectrometric Measurement of Intracellular Accumulated MG, Glyoxal, and 3-DG

The dicarbonyl metabolites glyoxal, MG, and 3-DG were determined by derivatization with 1,2-diaminobenzene (DB) and quantification of the resulting quinoxaline adducts was determined by stable isotopic dilution analysis liquid chromatography-tandem mass spectrometry (LC–MS/MS), as described elsewhere [23]. Briefly, 0.5 × 10^6^–1 × 10^6^ cultured cells were sonicated and subsequently mixed with 4% ice-cold trichloroacetic acid with 0.9% NaCl. Stable isotopic standards ([^13^C_3_]MG, [^13^C_2_]glyoxal and [^13^C_6_]3-DG)) were added and mixed, and samples were centrifuged. The supernatant was removed, sodium azide (0.3%) was added, and then finally 0.1 mM DB was added for derivatization over 4 h. Calibration standards containing 2 pmol of isotopic standards and 0–20 pmol of glyoxal, MG, and 3-DG were prepared and derivatized concurrently.

Samples were analyzed by LC–MS/MS with a C18 reverse-phase column (100 mm × 2.1 mm). The mobile phase was 0.1% TFA (trifluoroacetic acid) in water with a linear gradient of 0%–25% acetonitrile over 10 min. The quinoxaline adducts were detected by electrospray positive-ionization multiple reaction monitoring (MRM).

### 4.6. Statistics

Results of the experimental studies are reported as mean ± SEM and normalized to results of WT 25 mM + NT-siRNA, unless otherwise stated. Differences were analyzed by one-way ANOVA followed by Dunnett’s multiple comparison post-test using GraphPad Prism version 6.02 for Windows (GraphPad Software, San Diego, CA, USA). *p*-values < 0.05 were regarded as statistically significant. *N* describes independent biological experiments, whereas *n* accounts for the number of repetitions of measurements.

## 5. Conclusions

In summary, decreased expression of GLO1 in L6 cells in high glucose concentration induced dicarbonyl stress and increased collagen synthesis. This may relate to induction of fibrotic processes described in diabetic late complications [24] and probably to loss of functional contact of cells with ECM components by increased glycation with MG or the fibrotic process which is induced by collagen over-production, as described for cells cultured on MG-modified collagen matrices [32]. Increased expression of collagen 5 is interesting in this regards as it is critical for GLUT4 translocation to the cell surface and may mediate increased cell surface GLUT4 in response to GLO1 knockdown [34]. The observed difference between RNA and protein level of collagen 5 is of special interest and warrants further investigation.

## Abbreviations

GLO1	Glyoxalase 1
AGE	Advanced Glycation End Products
MG	Methylglyoxal
ECM	Extracellular Matrix
